# Supplementary material for: NF-κB Signaling Modulates miR-452-5p and miR-335-5p Expression to Functionally Decrease Epithelial Ovarian Cancer Progression in Tumor-Initiating Cells
Source: Int J Mol Sci. 2023 Apr 25;24(9):7826. doi: 10.3390/ijms24097826 (PMC10178396; doi:10.3390/ijms24097826)
Supplement: Supplementary file 1 [file ijms-24-07826-s001.zip › ijms-2280704-supplementary-caption.pdf]

Figure S1: Western Blot of OV90, ACI23, and OVCAR8 lysates silenced for either *RELA* or *RELB* were collected from either adh or sph conditions. Antibodies for RelA or RelB were used to confirm their respective silencing, with GAPDH as loading control.

Figure S2: Measuring expression of candidate miRNAs for further downstream study. A) The expression of miR-30a-3p, miR-200c-3p, and miR-155-5p was measured using cDNA generated from OV90 cells grown in adherent (ADH) or tumor initiating cell (TIC) spheroid conditions. B) The expression of miR-105-5p was measured across OV90 and ACI23 cells. C) The expression of miR-34a-5p expression was measured across OV90, ACI23, an OVCAR8 cells. Data was collected in triplicates.

Figure S3: Selection of inhibitor and mimic doses for hsa-miR-452-5p and hsa-miR-335-5p to use in functional assays. The expression of miR-452-5p and miR-335-5p by qRT-PCR of both inhibitor and mimic for hsa-miR-452-5p at various concentrations, (A,B). 90 pmol inhibitor and 0.1 pmol mimic were selected, and for miR-335-5p inhibitor and mimic doses of 90 pmol and 1pmol respectively, were selected (C,D). Experimental Ct values were normalized to hsa-miR-30e-5p and were plotted in triplicates.

Figure S4: EOC Functionality measured after miRNA modulation in cells silenced for *RELA* or *RELB*. A) Addition of NC or hsa-miR-452-5p mimic (452 mimic) at 0.1 pmol to OV90 spheres when *RELA* was silenced. B) Addition of NC or 452 mim in OVCAR8 spheres rescued the effect of reduced sphere formation when *RELB* was silenced. Spheres with an area greater than 1000 square microns were quantified. Data is plotted as mean with SEM. Analysis was done using an unpaired t-test. n.s, non-significant when compared to negative control (NC) C) Measured cell viability of OV90 spheres silenced for either *RELA* or *RELB* and transfected with a negative control (NC). Viability is measured relative to the shNeg NC control. D) Quantified percentages of ALDH+ OV90 cells with *RELA* or *RELB* silenced and transfected with a miR-335-5p mimic (335 mimic). Graphs represent data from three independent experiments (n=3) and is plotted to indicate mean with SD. Analysis was conducted using an unpaired t-test. n.s., non-significant when compared to control.

Figure S5: Measured cell viability of spheres after addition of a negative control (NC), hsa-miR-335-5p mimic (335 mim) or hsa-miR-335-5p inhibitor (335 Ib) in A) OV90 and B) OVCAR8 cells. Graphs represent mean with standard error of mean (SEM). Analysis was done using a two-way ANOVA and Dunnett's multiple comparisons test. * $p < 0.05$, ** $p < 0.01$, **** $p < 0.0001$, n.s., non-significant as compared to the negative control (NC) within each cell line (shNeg, shRelA, shRelB).

Figure S6: Colony formation ability after addition of miRNA inhibitors and mimics. Representative graphs showing number of colonies formed in *RELA* and *RELB* silenced OV90 and OVCAR8 cells after adding either an hsa-miR-452-5p inhibitor (A,B) or hsa-miR-335-5p mimic (C,D). Data is plotted as mean with SEM and analyzed using an unpaired t-test. * $p < 0.05$, ** $p < 0.01$, n.s., non-significant as compared to shNeg, shRelA, or shRelB negative control.

Table S1: List of miRNA Probes and their sequences.
