# Supplementary material for: NF-κB Signaling Modulates miR-452-5p and miR-335-5p Expression to Functionally Decrease Epithelial Ovarian Cancer Progression in Tumor-Initiating Cells
Source: Int J Mol Sci. 2023 Apr 25;24(9):7826. doi: 10.3390/ijms24097826 (PMC10178396; doi:10.3390/ijms24097826)

Supplementary Table S1: List of miRNA Probes and their sequences

Target Probe	Mature miRNA Sequence	Assay ID
hsa-miR-30e-5p	UGUAAACAUCCUUGACUGGAAG	479235_mir
hsa-miR-452-5p	AACUGUUUGCAGAGGAAACUGA	478109_mir
hsa-miR-335-5p	UCAAGAGCAAUAACGAAAAAUGU	478324_mir

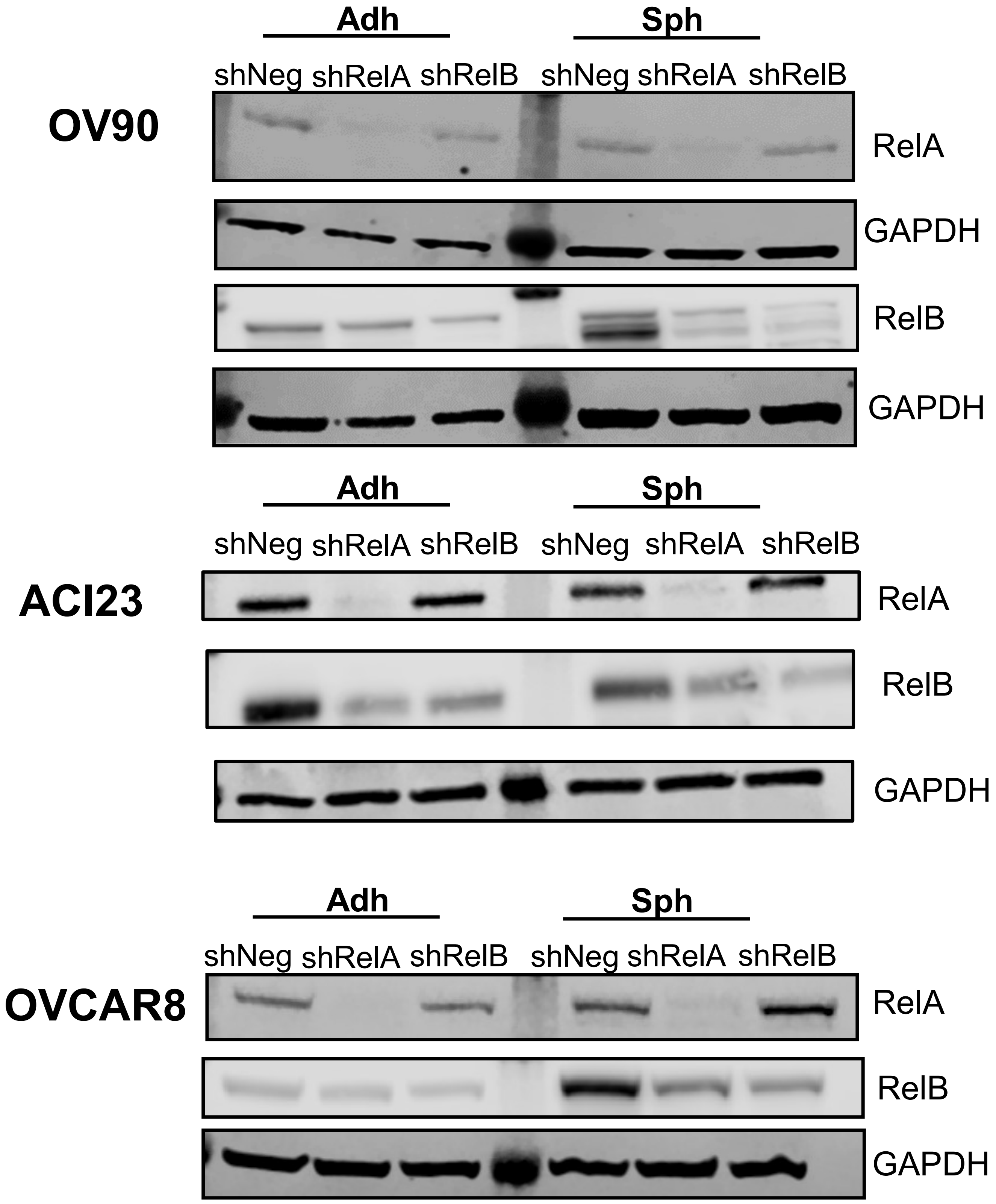
Adh
Sph
shNeg
shRelA
shRelB
shNeg
shRelA
shRelB
OV90
RelA
GAPDH
RelB
GAPDH
Adh
Sph
shNeg
shRelA
shRelB
shNeg
shRelA
shRelB
ACI23
RelA
RelB
GAPDH
Adh
Sph
shNeg
shRelA
shRelB
shNeg
shRelA
shRelB
OVCAR8
RelA
RelB
GAPDH

Supplementary Figure S2, Kamdar et al

A

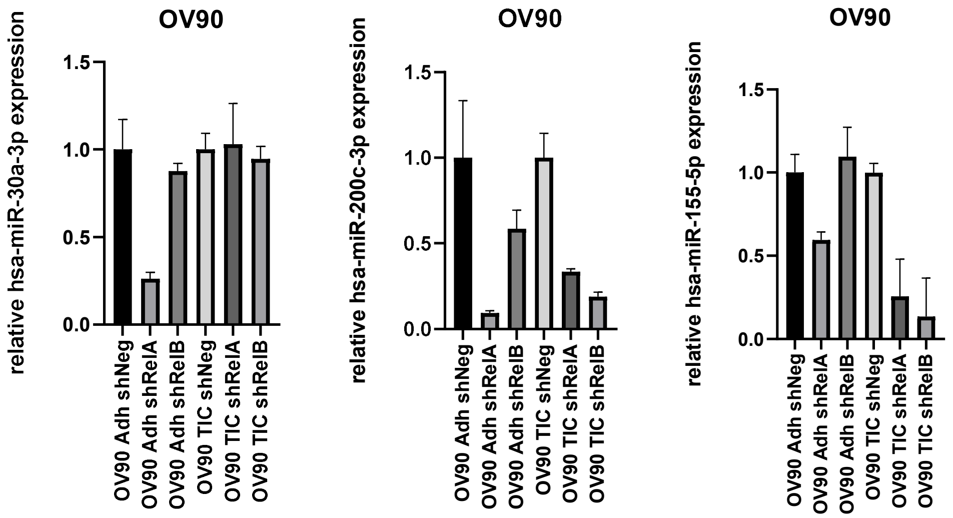

B

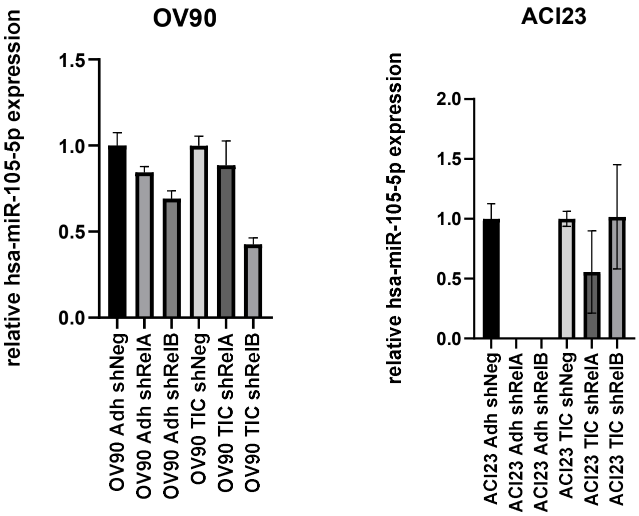

C

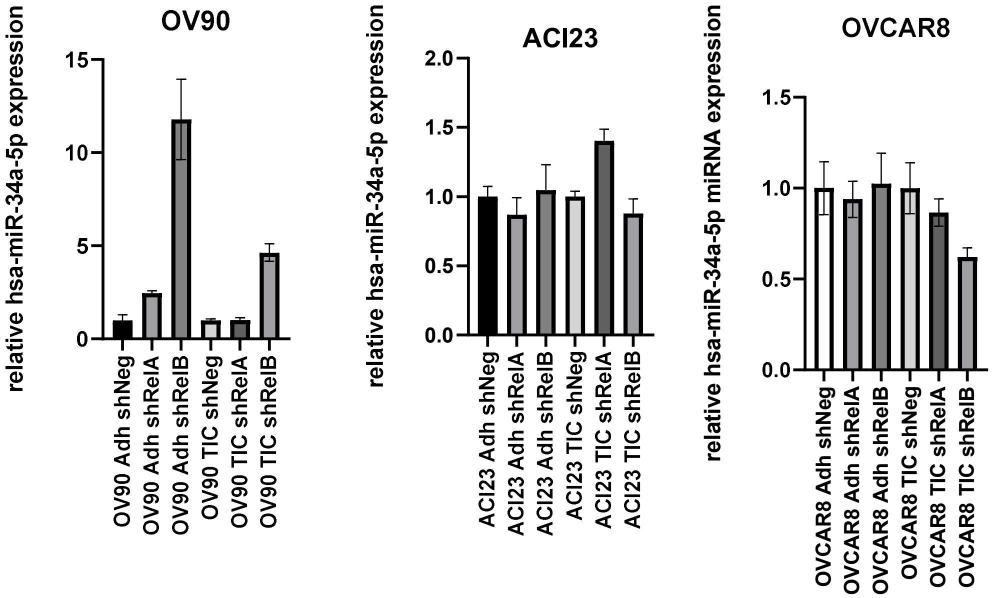

Supplementary Figure S3, Kamdar et al

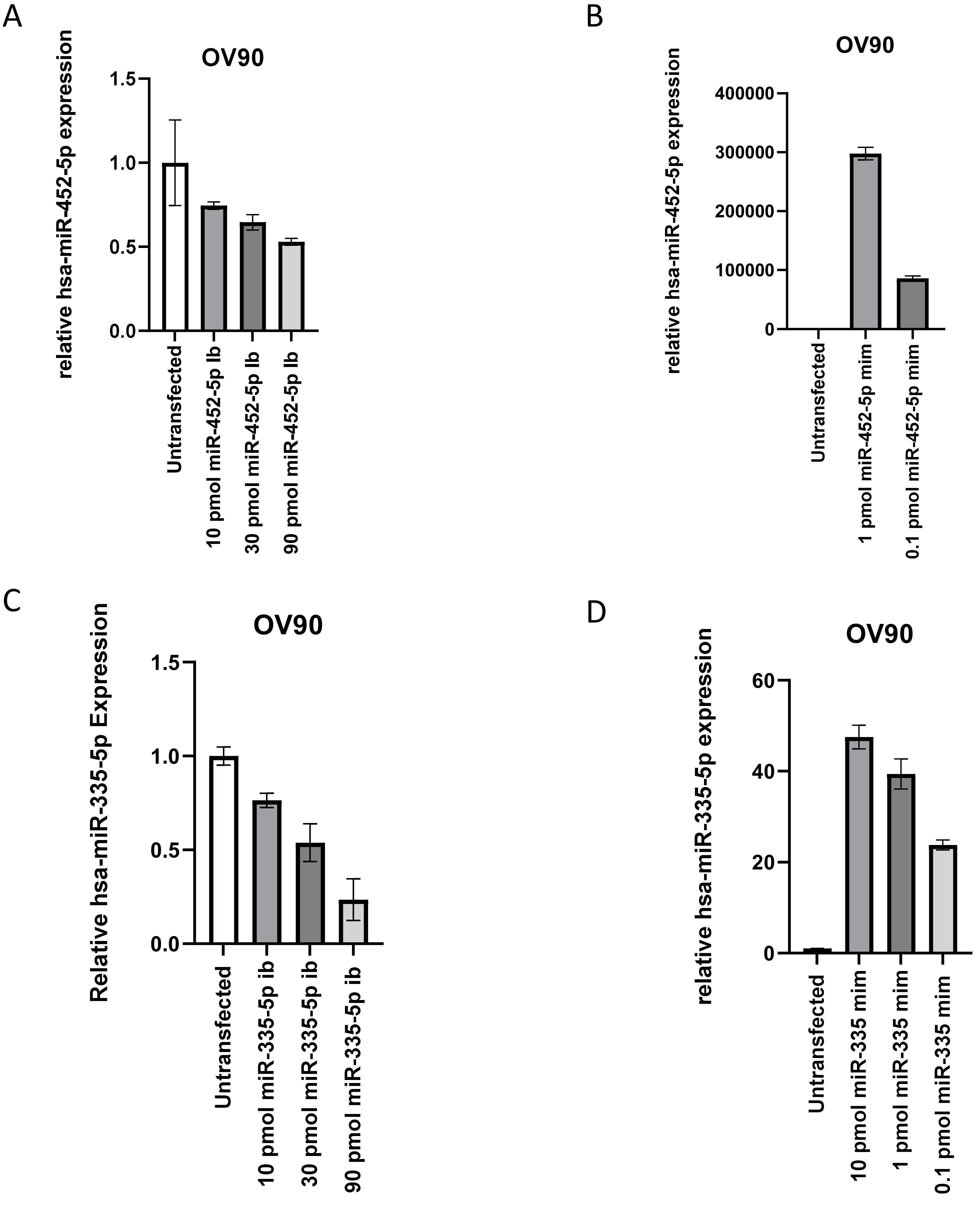

Supplementary Figure S4, Kamdar et al

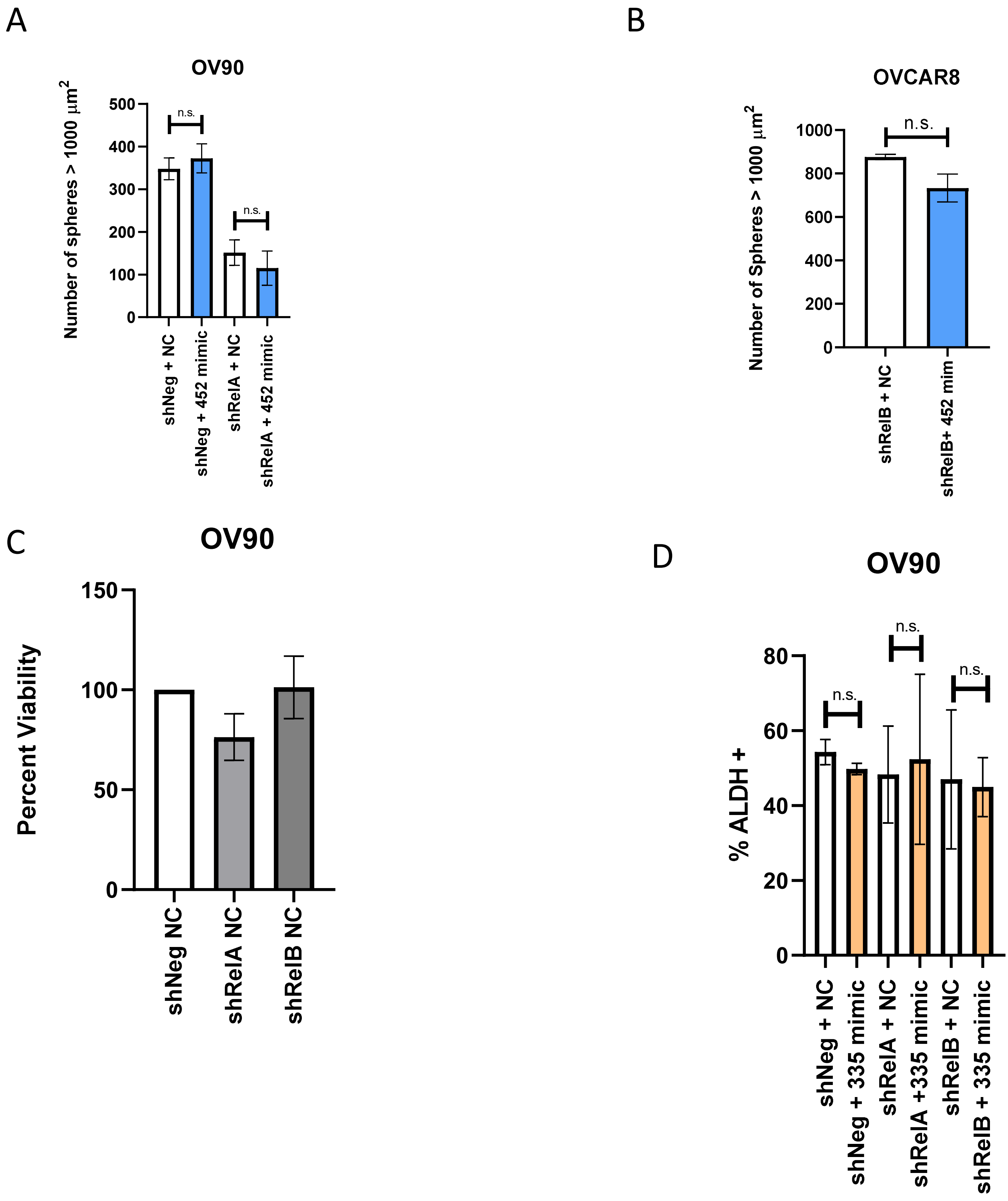

Supplementary Figure S5, Kamdar et al

A

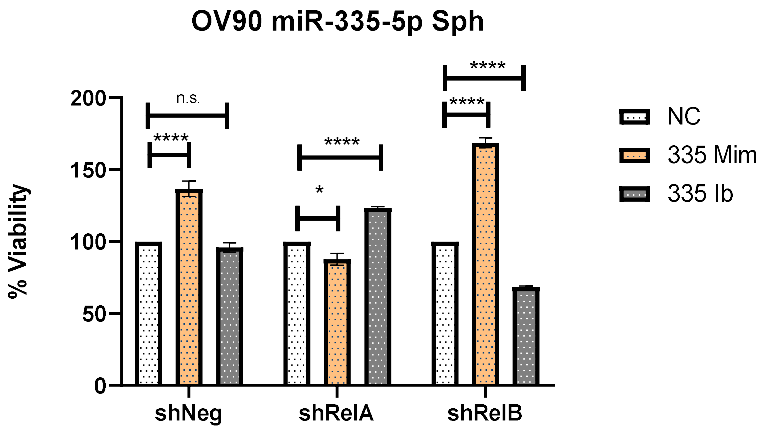

B

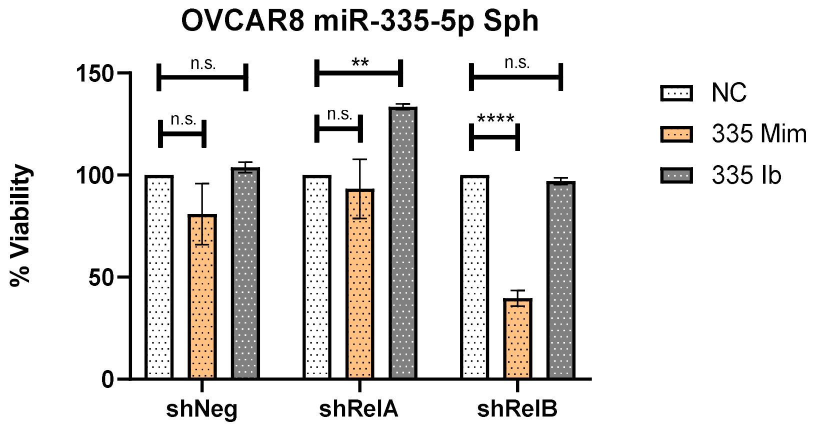

Supplementary Figure S6, Kamdar et al

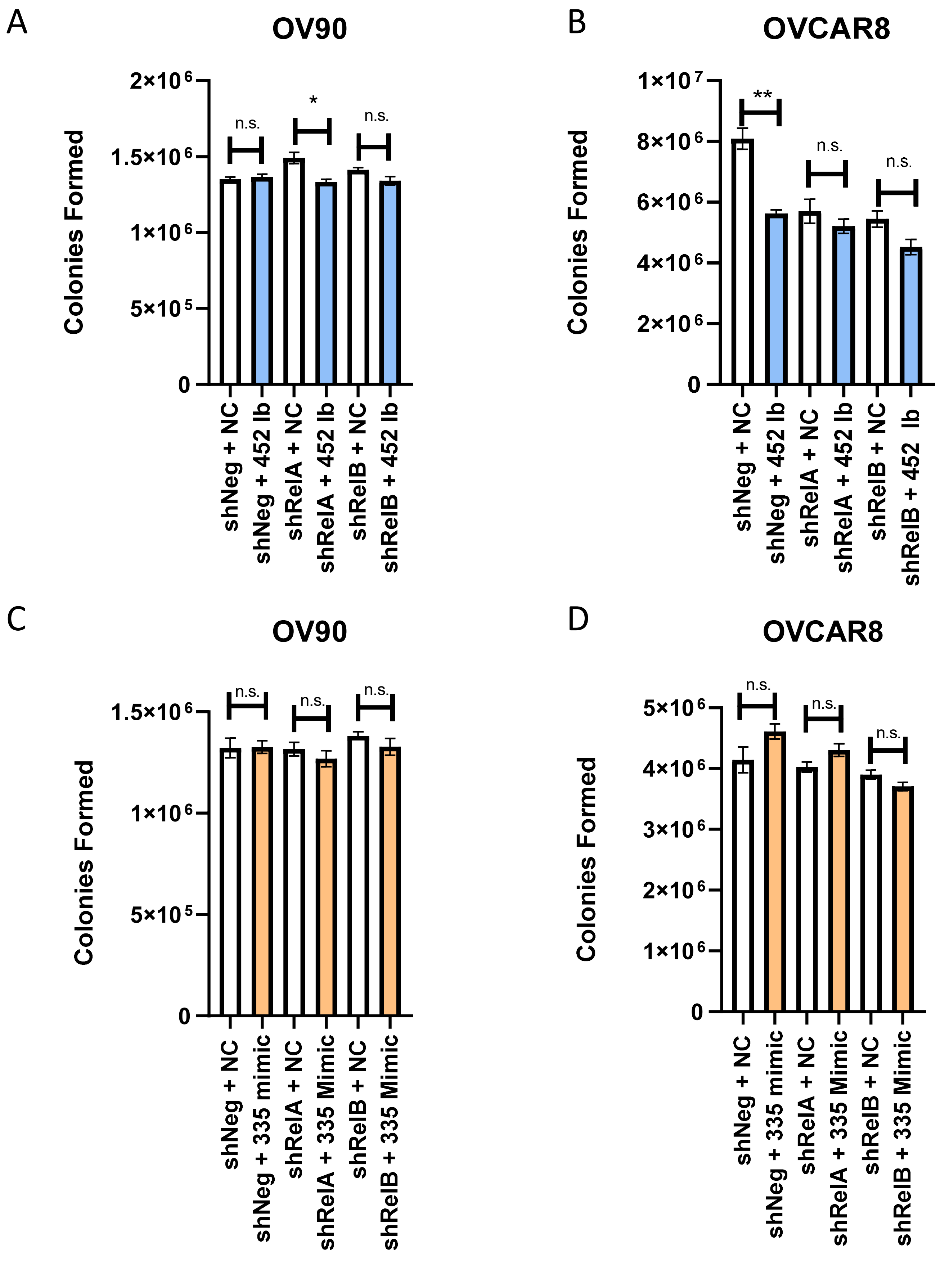

Supplement: Supplementary file 1 [file ijms-24-07826-s001.zip › ijms-2280704-supplementary.pdf]
